# Resorbable Bio‐Inductive Collagen Implant for Rotator Cuff Repair: What We Know, What We Need to Know, and the Path Forward

**DOI:** 10.1111/os.70141

**Published:** 2025-08-08

**Authors:** Jiaxin Tian, Fengxing Ding, Zhe Wang, Niu Muting, Chen Liu, Zipeng Ye, Huiang Chen, Caizhi Wu, Shaowei Yi, Yubo Fan, Jinzhong Zhao, Shiyi Cao, Bin Ma

**Affiliations:** ^1^ School of Biological Science and Medical Engineering Beihang University Beijing China; ^2^ Evidence‐Based Medicine Center School of Basic Medical Sciences, Lanzhou University Lanzhou China; ^3^ Research Center for Medical Device Regulatory Science Lanzhou University Lanzhou China; ^4^ School of Public Health Gansu University of Traditional Chinese Medicine Lanzhou China; ^5^ Department of Sports Medicine Shanghai Jiao Tong University Affiliated Sixth People's Hospital Shanghai China; ^6^ 1st School ofClinical Medicine, Lanzhou University Lanzhou China; ^7^ School of Public Health Wuhan China; ^8^ Industry Center for Evidence‐Based Researchand Evaluation Standards in Medical Devices Lanzhou China; ^9^ Research Unit of Evidence‐Based Evaluation and Guidelines, Chinese Academy of Medical Sciences (2021RU017), School of Basic Medical Sciences, Lanzhou University Lanzhou China; ^10^ Key Laboratory of Evidence Based Medicine of Gansu Province Lanzhou China

**Keywords:** bio‐inductive implant, meta‐analysis, rotator cuff, systematic review

## Abstract

**Objectives:**

Rotator cuff injuries are a leading cause of shoulder dysfunction, where bio‐inductive collagen implants have demonstrated promising results in promoting tendon regeneration and reducing retear rates. However, existing evidence lacks consistent evaluation across varying follow‐up durations, while the specific factors influencing their safety and effectiveness remain undetermined. This study aims to evaluate the quality of evidence regarding the safety, efficacy, and impact factors of applying the resorbable bio‐inductive collagen implant clinically to repair rotator cuff injuries.

**Methods:**

The study protocol was registered on PROSPERO (CRD42022367522). A systematic literature search of PubMed, Web of Science, Embase, and Cochrane Library (from inception to October 2024) for clinical studies on bio‐inductive collagen implants for rotator cuff repair. Two investigators independently screened studies, extracted data, and assessed quality (using RoB1 for RCTs, NOS for cohort studies and JBI critical appraisal tools for case series). Primary outcomes included postoperative tendon thickness, shoulder function scores (ASES/Constant), and re‐tear rates. Data were analyzed using random/fixed‐effects models to calculate mean differences (MDs) with 95% CIs, with subgroup analyses for tear type, patient age, and postoperative mobilization time. Statistical analyses were performed using Stata 17.0.

**Results:**

Seventeen studies were included. The meta‐analysis results showed that postoperative tendon thickness of the patients increased statistically compared with the baseline, at 3 months (MD = 2.22; 95% CI: 1.61, 2.83; *p* < 0.001), 6 months (MD = 2.30; 95% CI: 1.44, 3.16; *p* < 0.001), 12 months (MD = 2.15; 95% CI: 1.58, 2.72; *p* < 0.001), and 24 months (MD = 1.05; 95% CI: 0.02, 2.08; *p* = 0.045). Postoperative shoulder joint function improved significantly. The ASES score and Constant score of the patients were significantly higher than the baseline at 6 months (ASES: MD = 35.90; 95% CI: 32.97, 38.83; *p* < 0.001), 12 months (ASES: MD = 40.83; 95% CI: 37.56, 44.10; *p* < 0.001; Constant: MD = 28.59; 95% CI: 21.44, 35.74; *p* < 0.001), and 24 months (ASES: MD = 39.80; 95% CI: 31.32, 48.27; *p* < 0.001; Constant: MD = 32.84; 95% CI: 28.72, 36.97; *p* < 0.001).

**Conclusion:**

The bio‐inductive collagen implant is effective and safe for healing rotator cuff injuries. Patient age may be an important moderator affecting its efficacy. The impact of tear size and postoperative activities on efficacy needs to be further explored through in‐depth clinical studies.

## Graphical abstract

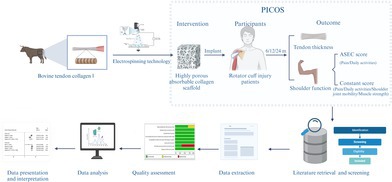

## 1 Introduction

The rotator cuff supports and stabilizes the scapulohumeral joint during shoulder movement and maintains the normal fulcrum relationship between the humeral head and the glenoid [1, 2, 3]. Rotator cuff injury is one of the common injuries of the shoulder joint. It is the main cause of adult shoulder dysfunction and pain, and profoundly affects sleep, work, activities, and psychosocial functions of patients [4]. Full‐thickness rotator cuff tears occur in 22% of those over 65 years of age [5] and in 31%–41% of those over 70 [6]. Approximately 250,000 rotator cuff repairs are performed annually in the United States (with an approximate cost of $6367 per patient) [7]. The cases of rotator cuff repairs increased by 141% between 1996 and 2006 alone [8].

Resorbable bio‐inductive collagen implant is a highly oriented and highly porous rotator cuff implant derived from bovine tendon type I collagen. It has been reported to have good mechanical properties, biocompatibility, and the ability to induce tissue regeneration, which can effectively promote tendon growth and vascularization at the tear site [7, 9]. A resorbable bio‐inductive collagen implant is put in place with a permanent bone anchor and an absorbable tendon anchor [10]. It helps create an environment conducive to wound healing and induces new soft tissue formation capable of integrating into native tissue [11]. It also helps share the mechanical load of the native tissue, thereby reducing the stress that may lead to microscopic tearing, injury, or tear propagation [11]. More importantly, clinical trials in recent years have shown that the use of resorbable bio‐inductive collagen implants might induce the formation of complete and mature tendon‐like tissue in partial and full‐thickness rotator cuff tears, significantly relieving pain caused by diseases, improving shoulder function, and reducing postoperative re‐tear rate [11, 12, 13, 14, 15, 16, 17]. At the same time, the use of resorbable bio‐inductive collagen implants to repair rotator cuff tears healed well; the postoperative fat infiltration rate was lower than that of equivalent arthroscopic rotator cuff repair, with no implant‐related (serious) adverse events [14, 18].

Recent systematic reviews have shown positive effects of bio‐inductive collagen patches in repairing rotator cuff injuries [19, 20, 21]. However, assessing the efficacy and safety of medical devices requires comprehensive analysis of data from different follow‐up time points. The Cochrane Handbook states that in long‐term studies [22], outcomes may be reported at multiple follow‐up periods. Analyzing data from a single time point may lead to reporting bias, while selecting the longest follow‐up from each study may cause inconsistencies between studies and increase heterogeneity. Therefore, in the absence of individual patient data, defining several outcomes based on different follow‐up periods and analyzing them separately may be the most appropriate approach currently. Moreover, multi‐timepoint assessments help observe the dynamic effects of implants on the human body. For example, tendon thickness, closely related to patient prognosis and treatment outcomes, reflects the sustainability of therapeutic effects through changes over time. Evaluating indicators such as tendon thickness at multiple time points provides deeper insights into the dynamic effects of implants and offers more comprehensive and reliable evidence for clinical decision‐making [23, 24, 25]. Furthermore, appropriate time points are crucial for evaluating the safety and efficacy of medical devices. Both China and the United States emphasize during the review process that follow‐up duration is a key factor in clinical trials for medical devices and one of the factors most likely to influence the interpretation of clinical outcomes [25, 26].

Additionally, several other factors should be considered when evaluating the efficacy and safety of the implant, such as rotator cuff tear type, patient age, and postoperative activity. Taking patient age as an example, older age is an independent risk factor for rotator cuff tears. Compared to younger patients, larger lesions, fatty degeneration, and poorer tendon quality often increase repair difficulty and hinder rotator cuff healing in older individuals [27]. Furthermore, age‐related comorbidities (such as osteoporosis and metabolic syndrome) may frequently affect surgical outcomes [27]. Therefore, assessing the efficacy of bio‐inductive collagen implants across different age groups helps clinicians make better individualized treatment decisions and conduct more targeted future research.

In the present study, we will conduct a retrospective analysis of published clinical studies on bio‐inductive collagen implants for the repair of rotator cuff injuries. The primary purpose is to evaluate the safety and efficacy of bio‐inductive collagen implants in repairing rotator cuff injuries, followed by the moderating factors affecting the efficacy of bio‐inductive collagen implants. The objective is to present reliable research evidence for the efficacy and clinical application of bio‐inductive collagen implants.

## 2 Method

### 2.1 Literature Search

All related articles about bio‐induced collagen implants for repairing rotator cuff injuries included in the databases of PubMed, Web of Science, Embase, and Cochrane Library were retrieved from the establishment of the database to September 2022. We have updated the search (search time updated to October 2024). In addition, we searched the reference lists of included studies. Details of the search strategies were presented in Appendix S1. The protocol was registered on PROSPERO (CRD42022367522).

### 2.2 Eligibility Criteria

Inclusion criteria: (1) Patients clinically diagnosed with rotator cuff injury. The inclusion of patients required clinical history and (or) diagnostic arthroscopy and (or) imaging data (including X‐ray, magnetic resonance arthrography (MRA), magnetic resonance imaging (MRI), or ultrasound [28, 29]) confirmed diagnosis as a rotator cuff tear. (2) The intervention of resorbable bio‐inductive collagen implants was adopted to repair rotator cuff injuries in the included studies, with no limit on the control measures. (3) The type of included research was clinical trial, with no limit on whether they were controlled trials. (4) Outcome measures included were postoperative tendon thickness (measured by MRA, MRI or ultrasound), shoulder joint function (evaluated by the American Shoulder and Elbow Surgeons Standardized Shoulder Assessment Form [ASES] score) or the Constant‐Murley score (Constant score), and the incidence of re‐tear (and/or repair failure) and implant‐related (serious) adverse events and non‐implant‐related (serious) adverse events (see Section 2.4). For postoperative tendon thickness and shoulder joint function, the measurement time points were the baseline, 6, 12, and 24 months after implantation, respectively.

Exclusion criteria: (1) case reports; (2) non‐Chinese and non‐English literature, and (3) research with no available original full text.

### 2.3 Study Selection

Two researchers (Jiaxin Tian and Fengxing Ding) independently used Endnote X9 for literature screening, data extraction, and cross‐checking according to the pre‐established inclusion and exclusion criteria. If opinions differ, a third researcher (Bin Ma) was consulted for judgment. In the initial screening, the titles and abstracts were reviewed according to the pre‐established inclusion/exclusion criteria, and then the full texts were reviewed after excluding irrelevant literature. Full‐text screening is detailed in Appendix S2.

### 2.4 Data Extraction and Management

Two researchers (Jiaxin Tian and Fengxing Ding) independently extracted data through a pre‐established full‐text data extraction table. The table contained the epidemiological characteristics of the included studies and the relevant features of diseases and interventions.

Epidemiological characteristics of the included studies comprised the first author, publication time, country/region, journal (and its 2021 impact factor), and product source (manufacturer).

Disease and intervention‐related characteristics of the included studies covered the diagnostic method, the classification method of rotator cuff tears (e.g., Ellman Classification [30], DeOrio and Cofield Classification [31], and Bateman Classification [32]), number of cases, follow‐up completion rate, age (mean ± standard deviation or range), gender, extent of rotator cuff tear (degree or type), surgical method, postoperative mobilization (early mobilization 0–2 W or delayed mobilization 4–8 W), and postoperative immobilization time (mean ± standard deviation or range). The primary outcomes were as follows: tendon thickness (measured by MRA, MRI, or ultrasound), shoulder function evaluation (evaluated by the ASES score or the Constant score), and retear rate and/or repair failure rate. Secondary outcomes included the incidence of (serious) adverse events related to collagen implants. For postoperative tendon thickness and shoulder function evaluation, the measurement time points were the baseline, 6, 12, and 24 months after implantation, respectively.

The following considerations should be noted. (1) After discussing with clinicians and experts in the field of biomaterials and evidence‐based medicine, it was decided that only (serious) adverse events related to collagen implants should be analyzed and discussed. Adverse events included infection, and serious adverse events included shock, sepsis, and death caused by infection, and local aseptic inflammation. If the investigator(s) stated in the study that the occurrence of a (serious) adverse event was related to the implant, then we considered the event to be related to the implant in our analysis. (2) After discussing with the above‐mentioned experts and combining the opinions of experts in the fields of epidemiology and statistics, we checked post‐procedure time points with important clinical significance for each judgment criterion. For details, see the outcome measures section of “Eligibility criteria.” (3) Definition of postoperative activities: the timing when physical therapy began to be supervised after surgery, with 0–2 weeks for early mobilization and 4–8 weeks for delayed mobilization [28].

Necessary data that could not be extracted from the literature were obtained directly by contacting the authors. When data were calculated or estimated (e.g., standard deviation calculated from standard error, *p* value and confidence interval [CI], or estimated from standard deviation in a graph or other experiments), they would be clearly labeled in Section 3.2.

### 2.5 Assessment of the Risk of Bias

The Risk of Bias 1.0 (RoB 1.0) [33] and Newcastle‐Ottawa Quality Scale (NOS) [33] were used to evaluate the quality of randomized controlled trials (RCTs) and cohort studies, respectively. The critical appraisal tools recommended by the Australian JBI Evidence‐Based Healthcare Center were used to evaluate the quality of the case series [34].

RoB1, developed by the Cochrane Collaboration, assesses six domains of risk of bias: random sequence generation, allocation concealment, blinding, incomplete outcome data, selective reporting, and other sources of bias. Each domain is rated as “high,” “low,” or “unclear,” indicating a high risk, low risk, or unclear risk of bias, respectively [35]. The NOS has eight items. Except for the item of “inter‐group comparability,” which was two points, all other items were 1 point, and the full score was nine points, with 0–4 being a low‐quality study and 5 to 9 being a high‐quality study [36]. The checklist for case series consists of 10 items, which aim to evaluate the quality of case reports from the aspects of participant inclusion, standardized measurement of the condition, clear reporting, and presentation of case data. Each item is judged as yes, no, unclear, or not applicable.

### 2.6 Statistical Analysis

For continuous variables such as tendon thickness (mm), ASES score, and Constant score, the mean difference (MD) was used as the effect size in this review. The heterogeneity among the included research results was analyzed by the *χ* ^2^ test (the level of significance *α* = 0.1), and the size of heterogeneity was quantitatively judged together with *I* ^2^. If there was no statistically significant heterogeneity among the results (*p* ≥ 0.10, *I* ^2^ ≤ 50%), a fixed effect model would be used for meta‐analysis. If there was statistically significant heterogeneity among the results (*p* < 0.10, *I* ^2^ > 50%), a random effect model would be used for meta‐analysis. Additionally, the source of heterogeneity would be analyzed by means of subgroup analysis or sensitivity analysis. Sensitivity analysis was conducted by excluding studies with small samples and with only estimates of SD (standard deviation) to assess their impact on the pooled results.

This review was intended to carry out subgroup analysis for the following factors: (1) different tear types (partial thickness vs. full thickness rotator cuff tear); (2) different ages of patients (< 60 years old vs. ≥ 60 years old); (3) different postoperative motion timelines (early mobilization 0–2 W vs. delayed mobilization 4–8 W) [28].

When more than 10 studies were available for an outcome measure, publication bias was assessed using funnel plots and Egger's regression test. All statistical analyses were conducted using Stata software (version 17.0; Stata Corp LLC, USA), with a two‐sided *p* value < 0.05 considered statistically significant.

## 3 Results

### 3.1 Study Selection

**FIGURE 1:**
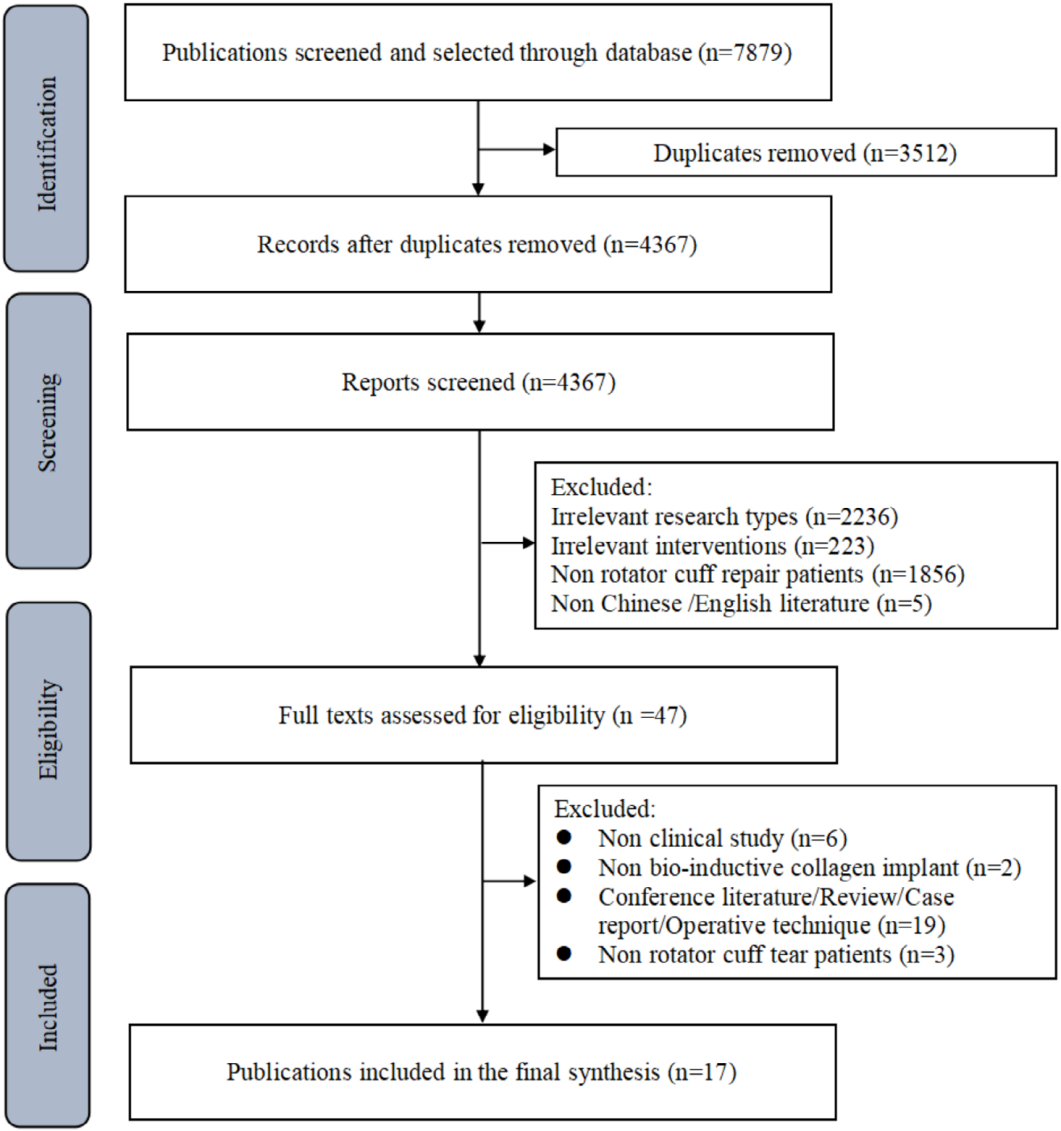
Flowchart of literature selection.

A total of 7879 documents were retrieved from databases, from which 3512 duplicate publications were removed and 4320 articles were excluded. After reviewing full‐text publications, 20 articles [9, 11, 13, 15, 16, 17, 18, 37, 38, 39, 40, 41] were included in this systematic review (Figure 1), involving a total of 17 studies.

### 3.2 Study Characteristics

A total of 17 clinical studies were included, including two RCTs [42, 43], four cohort studies [44, 45, 46], and 11 case series [9, 15, 17, 18, 39, 40, 41, 42, 43, 44, 45, 46, 47, 48, 49]. The earliest study was published in 2015. Most studies were conducted in the United States [9, 13, 17, 18, 39, 40, 44, 45, 47, 48], followed by Spain [41, 42, 43, 49] and Australia [15, 37, 46].

A total of 1329 subjects were included in the 17 studies (Table 1). The sample size ranged from 13 to 272, and the average age of the participants was 52–61 years old. All included studies except for one study [45] recruited mixed‐sex participants (*n* = 1255), including 733 males (58.4%) and 522 females (41.6%). The average follow‐up time ranged from 12 to 60.3 months, and the follow‐up completion rate of all studies was above 80%. The average postoperative suspension fixation time was up to 8 weeks [18], and the shortest was no more than 1 week [15]. Regarding the extent of rotator cuff tears [30], eight studies recruited patients with full‐thickness tears [18, 40, 42, 43, 44, 45, 46, 47], five studies recruited patients with partial‐thickness tears [9, 15, 37, 39, 48], and four studies recruited both patients with full‐thickness tears and those with partial‐thickness tears [13, 17, 41, 49].

**TABLE 1:** Summary of the included studies.

Study	Country	Study design	Indications, Yes or No/*n*		Classification method	Sample size	Follow‐up period, month (mean ± sd or range)	Follow‐up completion rate (%)	Gender, male/female	Sling time, week (mean ± sd or range)	Post‐op mobilization timing	Age, (mean ± sd or range)	Retear/failure to heal (*n*, %)
			Full‐thickness rotator cuff tear	Partial‐thickness rotator cuff tear									
Bokor [13]	Australia	Case series	Yes/8	Yes/1	NR	9	25.8 (24.5–30.4)	100.0	6/3	During the first 6 weeks	During the first 6 weeks	56.4 (50–66)	0
Bokor [15]^a^	Australia	Case series	No	Yes	Ellman	13	60.3 (59.1–62.1)	100.0	8/5	1 (NR)	Maximum of 1 week	54.0 ± 8.3	NR
Bushnell [9]	USA	Case series	No	Yes	Ellman	272	12.7 ± 2.8	83.5	146/126	2.8 ± 2.5	NR	52.1 ± 10.0	3, 1.1
Bushnell [47]	USA	Case series	Yes	No	Cofield	115	25.2 (18–34.8)	90.4	76/39	5.5 ± 2.6	NR	60.4 ± 8.0	3, 46.1
Camacho‐Chacon [41]^b^	Spain	Case series	Yes/12	Yes/18	Ellman; Bateman	30	12 (NR)	100.0	14/16	4 (NR)	NR	56.5 (35–74)	NR
McIntyre [17]^c^	USA	Case series	Yes/83	Yes/90	Ellman; Cofield	173	12.7 (12.0–17.2)	85.0	98/75	3.8 ± 2.9	UC	54.2 ± 9.8	4, 2.0
McIntyre [40]	USA	Case series	Yes	No	Cofield	210	12.6 (3.3–25.8)	91.4	131/79	5.2 ± 2.4	NR	57.5 ± 8.9	11, 5.2
Schlegel [39]	USA	Case series	No	Yes	Ellman	33	25.5 (21.3–34.9)	93.9	19/14	3.5 ± 2.0	NR	54.0 (33–74)	NR
Thon [18]	USA	Case series	Yes	No	Cofield	23	24 (NR)	100.0	15/8	6–8	NR	57.9 (32–71)	2, 8.7
Yeazell [37]	USA	Case series	No	Yes	NR	64	At least 6 months of follow‐up	100.0	36/28	For 3 weeks	UC	53.5 ± 10.2	NR
Ruiz Ibán [42]^d^	Spain	RCT	Yes	No	NR	124	12 (NR)	98.4	61/63	6 (NR)	Passive range of motion exercises were allowed after the first 3 weeks	57.7 ± 7.7	5, 8.3
Camacho Chacón [43]^e^	Spain	RCT	Yes	No	Bateman; Goutallier	60	24 (NR)	100.0	30/30	2 (NR)	NR	55.3 ± 6.6	0
Dai [48]^f^	USA	Case series	No	Yes	NR	30	19.1 (NR)	80.0	19/5	Discontinue the use of the sling within the first week	Within the first week	54.5 ± 11.6	1, 3.3
Zhang [44]	USA	Cohort study	Yes	No	Goutallier	48	At minimum 6 months	100.0	36/12	Additional 3 to 4 weeks of intermittent sling use outside the home	Active assisted range of motion exercises were initiated at 4 weeks after surgery	61 (40–77)	9, 37.5
Tisherman [45]	USA	Cohort study	Yes	No	Goutallier	38	42 ± 20.4	100.0	NR	NR	NR	60.3 ± 8.4	6, 31.6
Yatseta Yatseta and Oleksiy [49]	Spain	Case series	Yes/NR	Yes/NR	NR	6	23.4 (NR)	100.0	1/5	4 (NR)	4w: passive mobilization	47.3 ± 10.0	NR
Ting [46]	Australia	Cohort study	Yes	No	NR	51	23.4 (6–120)	100.0	37/14	6 (NR)	Day 1	56 (38–69)	9, 47.4

Abbreviation: NR, not reported.

^a^ The average follow‐up time of 11 participants was 60.3 months, the longest postoperative suspension time was 1 week, and the age was 54.0 ± 8.3 years old.

^b^ This study was followed for 12 months, but the average length of follow‐up was not reported. The postoperative suspension time was 4 weeks for patients with full‐thickness tears, and the immobilization is released as soon as possible for patients with partial‐thickness tears.

^c^ There were 203 participants in this study, but only 173 patients completed the one‐year follow‐up. The follow‐up completion rate was 85%. Due to the fact that this study did not report the number of male and female participants at baseline and only reported relevant information for the first year after surgery, for the sake of description, we used data from the first year after surgery to calculate the proportion of male and female participants.

^d^ This study was followed for 12 months, but the average length of follow‐up was not reported.

^e^ This study was followed for 24 months, but the average length of follow‐up was not reported.

^f^ This study included a total of 30 patients, but only 24 patients completed follow‐up. The author only reported the gender participation of these 24 patients.

Five studies qualified for meta‐analysis [9, 15, 39, 40, 47]. The results of the remaining 12 studies [13, 17, 18, 37, 41, 42, 43, 44, 45, 46, 48, 49] were not available due to unreported or incomplete reporting, and necessary outcome measures could not be obtained from the author either. Only descriptive analysis was given for this part of the review (Table 2).

**TABLE 2:** Relevant outcome indicators of the included studies.

Study	Tendon thickness (mean ± sd, MRI)					ASES score (mean ± sd)				Constant score (mean ± sd)				Adverse event (implant related)
	Baseline	Post‐op 3 m	Post‐op 6 m	Post‐op 12 m	Post‐op 24 m	Baseline	Post‐op 6 m	Post‐op 12 m	Post‐op 24 m	Baseline	Post‐op 6 m	Post‐op 12 m	Post‐op 24 m	
Bokor [15]^a^	4.28 ± 1.15	6.40 ± 1.20	6.58 ± 1.08	6.53 ± 1.36	5.91 ± 0.76	51.44 ± 24.60	NR	NR	81.63 ± 26.97	66.14 ± 25.55	NR	NR	85.30 ± 18.93	No
Bushnell [9]	NR	NR	NR	NR	NR	46.80 ± 18.2	82.70 ± 18.40	88.10 ± 17.90	NR	NR	NR	NR	NR	No
Bushnell [47]^b^	4.04 ± 1.49	NR	NR	NR	4.62 ± 1.37	50.59 ± 18.63	NR	93.79 ± 12.26	95.88 ± 11.42	50.07 ± 17.33	NR	81.77 ± 11.29	83.97 ± 9.46	Infection; Intermittent pain in the treated shoulder
Camacho‐Chacon [41]	UC	NR	UC	NR	NR	UC	UC	UC	NR	UC	UC	UC	NR	No
McIntyre [17]	NR	NR	NR	NR	NR	UC	UC	UC	NR	NR	NR	NR	NR	Postoperative infection
McIntyre [40]	NR	NR	NR	NR	NR	46.20 ± 19.80	82.10 ± 18.5	87.80 ± 18.40	NR	NR	NR	NR	NR	Infection
Schlegel [39]^b^, ^c^	3.10 ± 1.72	5.40 ± 1.72	UC	5.20 ± 1.15	UC	57.00 ± 18.38	NR	89.10 ± 15.84	93.69 ± 12.69	57.10 ± 16.08	NR	81.40 ± 12.45	90.05 ± 9.03	Increased accumulation of prominent subacromial fluid; Pain
Thon [18]	NR	NR	NR	NR	5.13 ± 1.06	NR	NR	NR	82.87 ± 16.68	NR	NR	NR	NR	No
Ruiz Ibán [42]	UC	UC	UC	UC	UC	44.6 ± 16.4	UC	UC	UC	UC	UC	UC	UC	Superficial infection Deep infection
Camacho Chacón [43]	UC	UC	UC	UC	UC	UC	UC	UC	UC	UC	UC	UC	UC	NR
Dai [48]	UC	UC	UC	UC	NR	UC	UC	UC	UC	NR	NR	NR	NR	No
Zhang [44]^d^	NR	NR	NR	NR	NR	38 ± 6.4	84 ± 3.8	NR	NR	NR	NR	NR	NR	No
Tisherman [45]^e^	NR	NR	NR	NR	NR	39.7 ± 16.4	NR	NR	64.6 ± 20.1	NR	NR	NR	NR	NR
Yatseta Yatseta and Oleksiy [49]	NR	NR	NR	NR	NR	15 ± 0.9	NR	NR	NR	51.7 ± 2.0	NR	NR	NR	No
Ting [46]	NR	NR	NR	NR	NR	NR	NR	NR	NR	NR	NR	NR	NR	No

Abbreviations: NR, not reported; UC, unclear.

^a^ Standard deviation estimated from images (by GetData Graph Digitizer 2.20).

^b^ Standard deviation estimated from subgroups.

^c^ Standard deviation estimated from standard error.

^d^ ASES was evaluated at least 6 months post‐op.

^e^ ASES was evaluated at 3.5 ± 1.7 years post‐op.

### 3.3 Quality Assessment and Sensitivity Analysis

**FIGURE 2:**
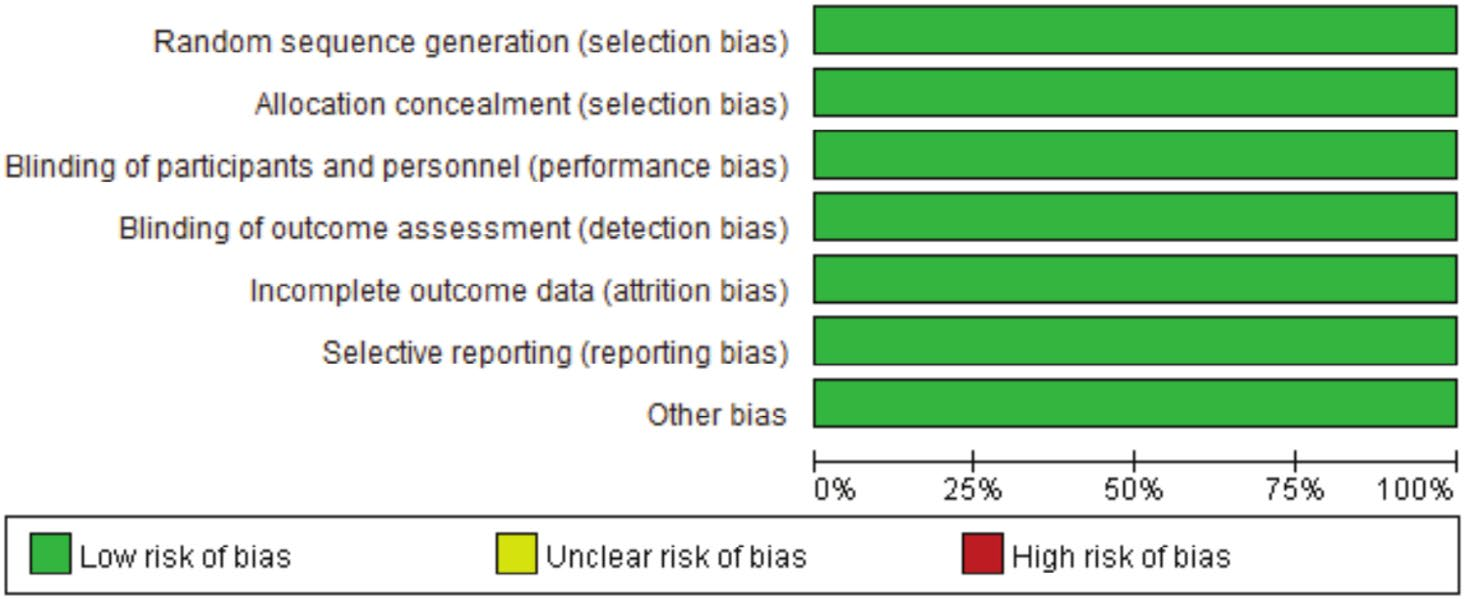
Quality evaluation results for the RCTs.

**FIGURE 3:**
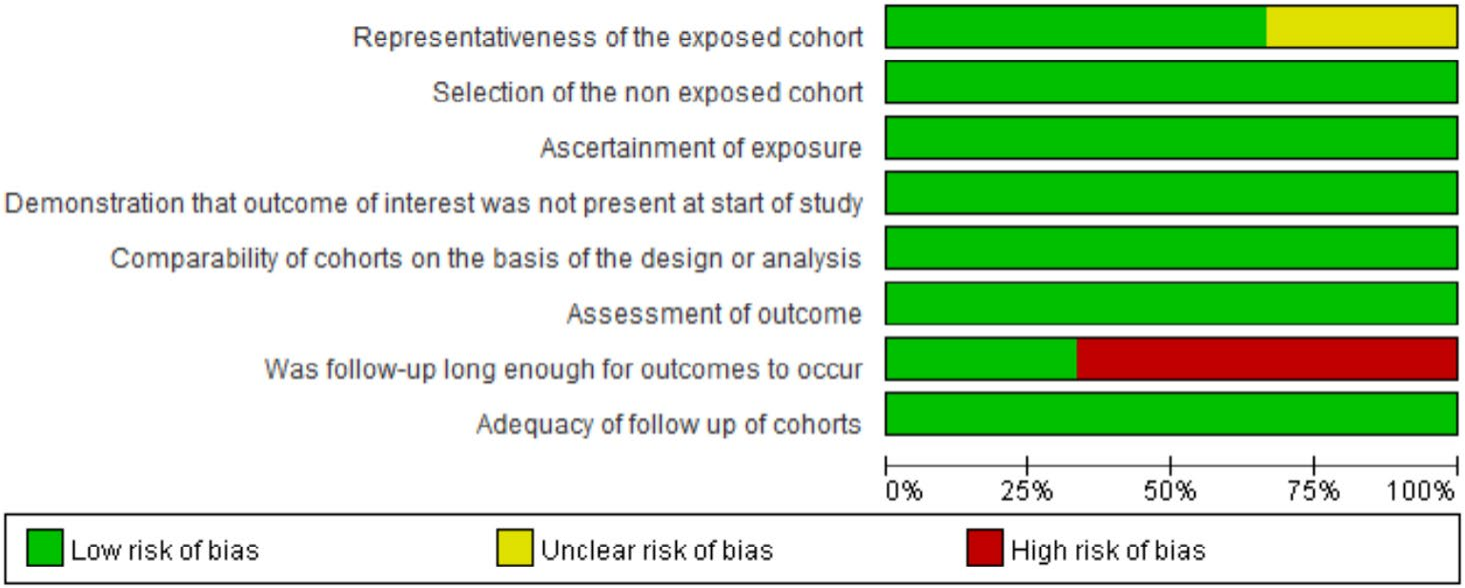
Quality evaluation results for the cohort studies.

**FIGURE 4:**
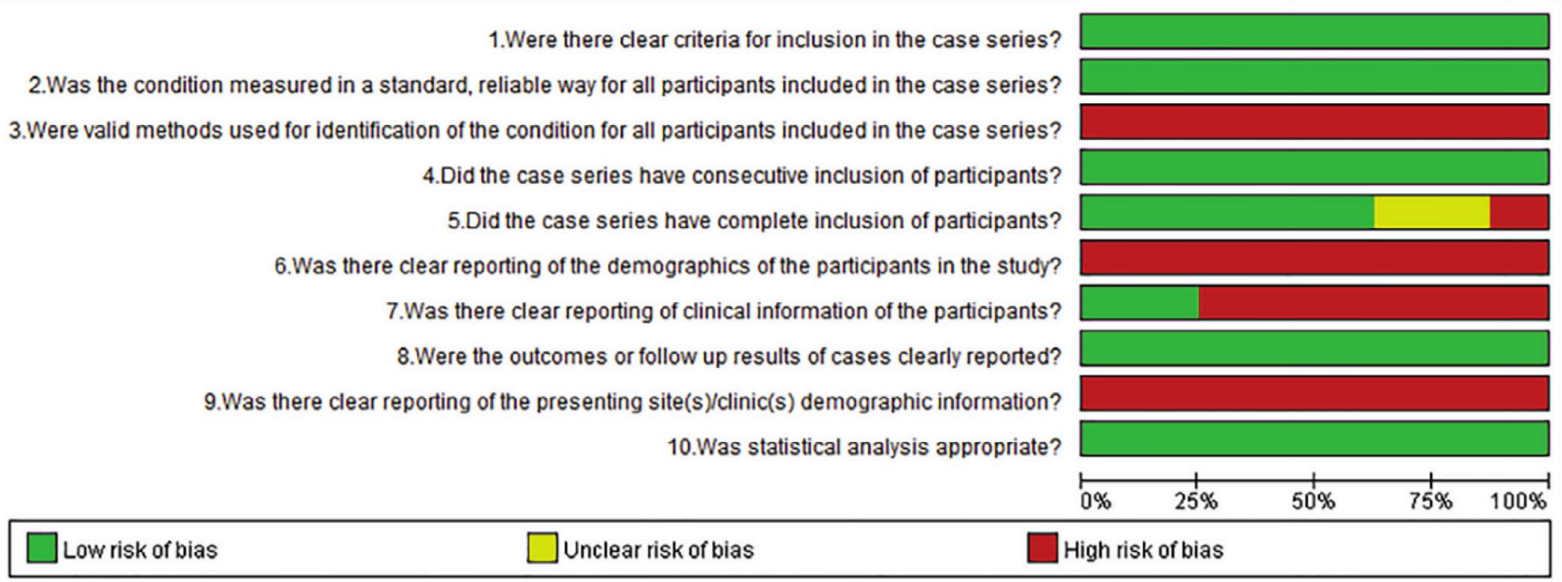
Quality evaluation results for the case series.

Figures 2, 3, 4 show the results of the risk of bias assessment of RCTs (Figure 2), cohort studies (Figure 3), and case series (Figure 4), respectively. The overall quality of the RCTs was good. All the quality assessment results of the four included cohort studies were seven, indicating high‐quality studies. The overall quality of the case series was moderate. Under the categories of clear criteria for inclusion, standard measurement of the condition, consecutive inclusion of patients, clear reporting of outcomes or follow‐up results, and appropriate statistical analysis, all case series were at low risk of bias. However, under the categories of valid methods used for identification of the condition, clear reporting of the presenting site(s)/clinic(s) demographic information, and clear reporting of the demographics of the participants, there was a high risk of bias for the case series. Two studies had incomplete inclusion of participants [11, 41]. Specifically, it could not be known whether the patients came from a single center or multiple centers; hence, the comprehensiveness of the inclusion of participants could not be determined. See Appendix S3 for the detailed risk of bias assessment results of all studies.

### 3.4 Results of the Meta‐Analysis

#### 3.4.1 Postoperative Tendon Thickness

**FIGURE 5:**
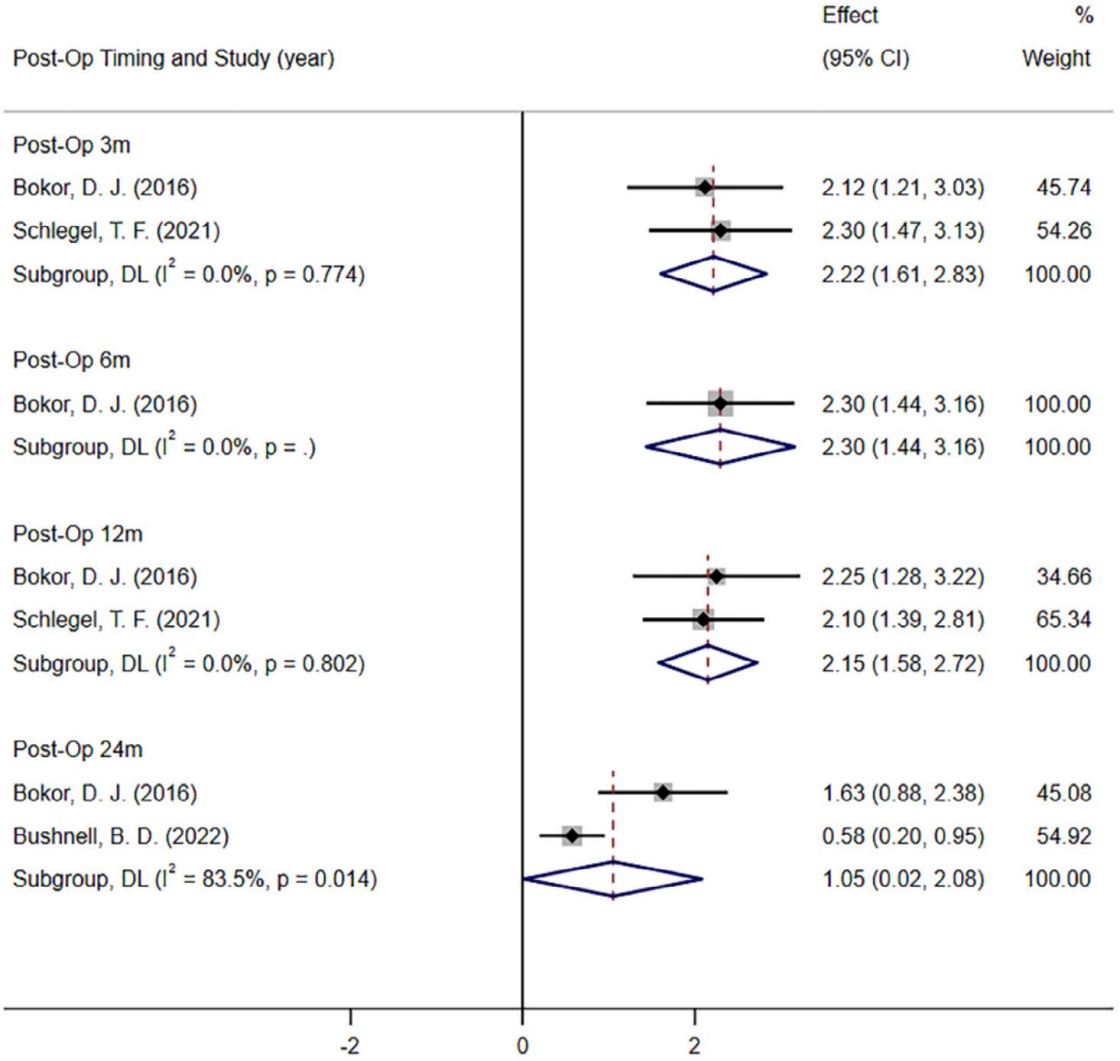
Forest plot of the tendon thickness between baseline and post‐operation.

Three studies reported postoperative tendon thickness (measured by MRI) in patients at 3 months [11, 39], 6 months [11], 12 months [11, 39], and 24 months [11, 47] (Figure 5). Meta‐analysis results showed that the postoperative tendon thickness was significantly higher than the baseline at 3 months (MD = 2.22; 95% CI: 1.61, 2.83; *I* ^2^ = 0.0%; *p* < 0.001), 6 months (MD = 2.30; 95% CI: 1.44, 3.16; *I* ^2^ = 0.0%; *p* < 0.001), 12 months (MD = 2.15; 95% CI: 1.58, 2.72; *I* ^2^ = 0.0%; *p* < 0.001) and 24 months (MD = 1.05; 95% CI: 0.02, 2.08; *I* ^2^ = 83.5%; *p* = 0.045).

#### 3.4.2 Postoperative Shoulder Function

**FIGURE 6:**
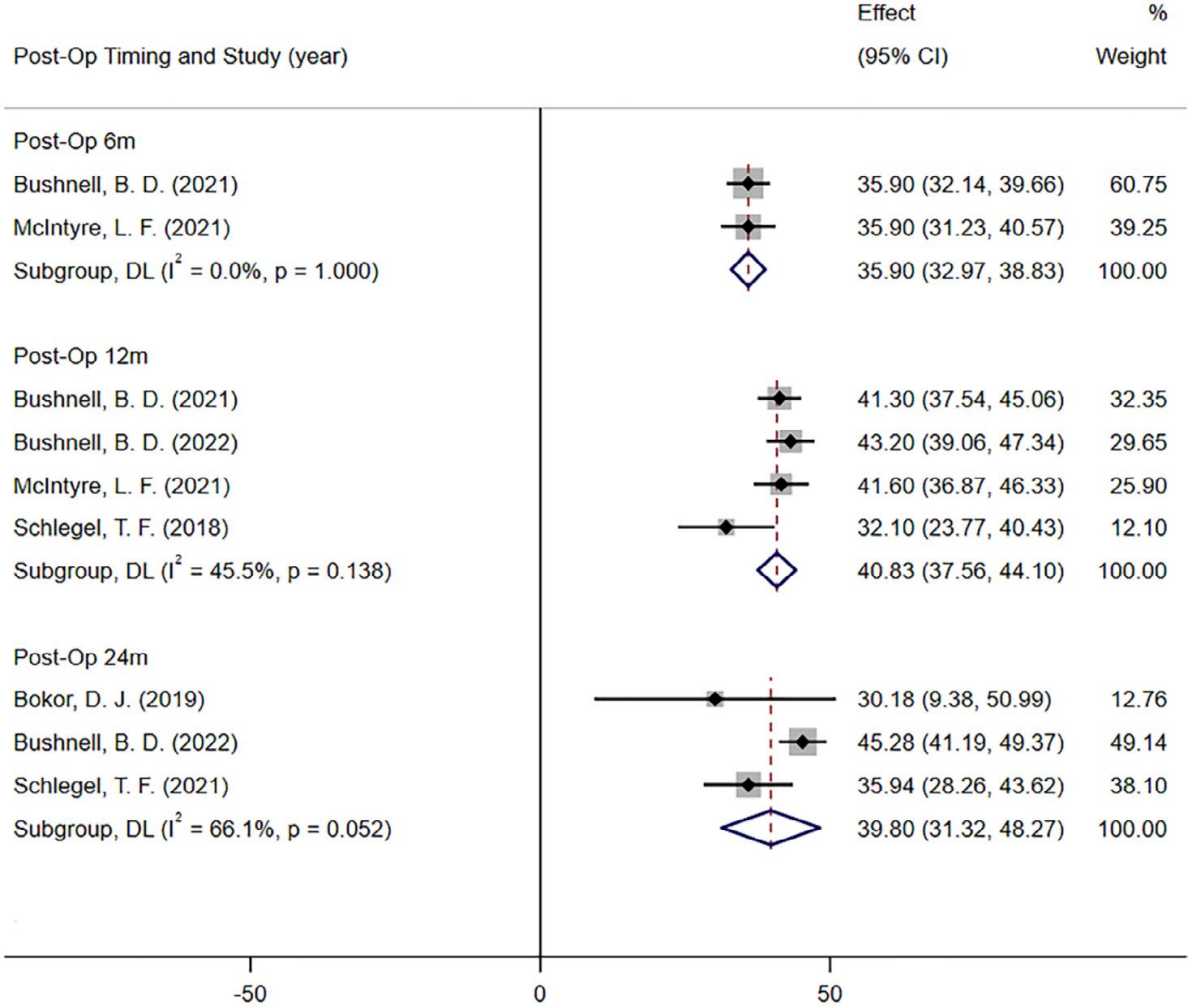
Forest plot of the ASES score between baseline and post‐operation (ASES score core 0–100, 100 is best).

Five studies reported postoperative shoulder function (all based on the ASES score) at 6 [9, 40], 12 [9, 38, 40, 47], and 24 months [15, 39, 47] (Figure 6). The results of the meta‐analysis showed that the postoperative ASES scores of patients were all significantly higher than the baseline at 6 months (MD = 35.90; 95% CI: 32.97, 38.83; *I* ^2^ = 0.0%; *p* < 0.001), 12 months (MD = 40.83; 95% CI: 37.56, 44.10; *I* ^2^ = 45.5%; *p* < 0.001) and 24 months (MD =39.80; 95% CI: 31.32, 48.27; *I* ^2^ = 66.1%; *p* < 0.001).

**FIGURE 7:**
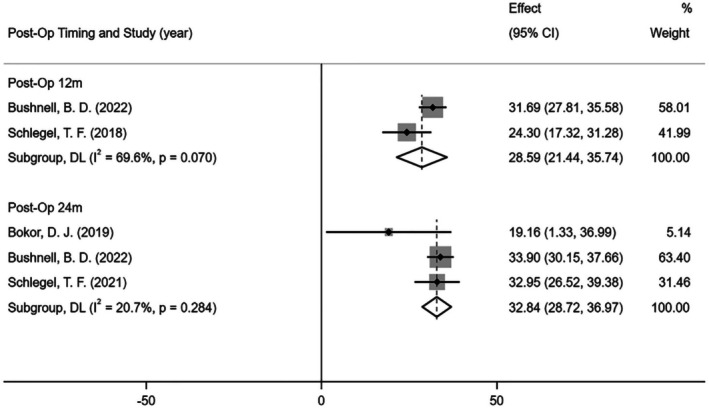
Forest plot of the Constant score between baseline and post‐operation (Constant score 0–100, 100 is best).

Three studies reported postoperative shoulder function (all based on the Constant score) at 12 [39, 47] and 24 months [15, 39, 47] (Figure 7). The results of the meta‐analysis showed that the patients' postoperative Constant scores all increased significantly compared with the baseline at 12 months (MD = 28.59; 95% CI: 21.44, 35.74; *I* ^2^ = 69.6%; *p* < 0.001) and 24 months (MD = 32.84; 95% CI: 28.72, 36.97; *I* ^2^ = 20.7%; *p* < 0.001).

### 3.5 Subgroup Analysis

**FIGURE 8:**
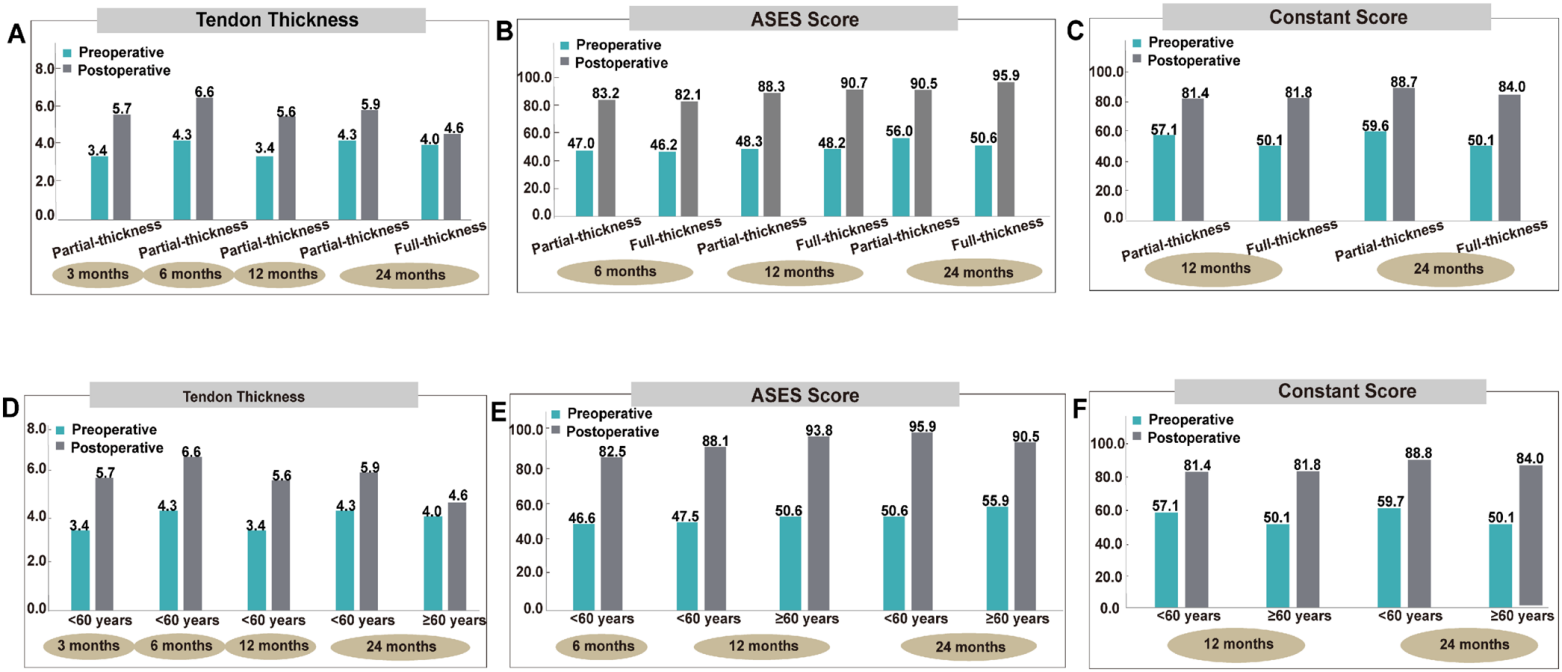
Subgroup analysis (A–C: Stratified by tear severity; D–F: Stratified by patient age).

Patients with different degrees of rotator cuff tears showed variations in postoperative recovery outcomes. As shown in Figure 8A–C, tendon thickness increased from 5.7 mm at 3 months to 6.6 mm at 6 months postoperatively, then decreased to 5.6 mm at 12 months. This observation may be related to data heterogeneity: on one hand, the data came from different studies with varying baseline characteristics among included patients (e.g., a higher proportion of massive tears compared to moderate tears), which may have influenced the results; on the other hand, the limited number of included studies and relatively small sample sizes may have reduced the stability of statistical results, making it difficult to accurately reflect the true changes in tendon thickness. At 24 months, patients with partial‐thickness tears showed greater average tendon thickness than those with full‐thickness tears (5.9 mm vs. 4.6 mm). In terms of postoperative functional assessment, preoperative ASES scores showed no significant differences between the two groups. At 6 months postoperatively, the partial‐thickness tear group showed slightly greater improvement than the full‐thickness tear group (83.2 vs. 82.1), but at 12 and 24 months, the full‐thickness tear group demonstrated more significant improvement. Constant scores showed lower preoperative values in the full‐thickness tear group. At 12 months, scores were similar between the full‐thickness and partial‐thickness tear groups (81.8 vs. 81.4), but by 24 months, the partial‐thickness tear group maintained better recovery outcomes (88.7 vs. 84.0).

Patients of different ages showed variations in postoperative recovery outcomes. As shown in Figure 8D–F, patients ≥ 60 years old had significantly lower tendon thickness at 24 months compared to those < 60 years old (4.6 mm vs. 5.9 mm). For ASES scores, among patients < 60 years old, the scores showed progressive improvement over time. At 12 months, the ≥ 60 group had higher scores than the < 60 group (93.8 vs. 88.1). It should be noted that data for ≥ 60 patients came from only one study [47], which only included patients with moderate and massive tears. By 24 months, ASES scores in ≥ 60 patients were significantly lower than in < 60 patients (90.5 vs. 95.9). For Constant scores, the two age groups had highly similar scores at 12 months (81.4 vs. 81.8), but by 24 months, the < 60 group showed clinically superior outcomes (88.8 vs. 84.0). Comprehensive evaluation results indicated that patients < 60 years old demonstrated better clinical outcomes in both functional recovery and structural healing after rotator cuff repair.

Among studies reporting postoperative mobilization time, only one study's data met the requirements for meta‐analysis [15], though seven studies reported relevant outcome measures. To better evaluate the effects of different postoperative mobilization time on collagen‐induced implantation for rotator cuff repair, we fully considered these results when qualitatively describing relevant subgroups, with four studies [15, 42, 46, 48] classified as the early mobilization group and three studies [13, 44, 49] as the delayed mobilization group.

In terms of tendon thickness, results showed that the delayed mobilization group had better outcomes than the early mobilization group at 3, 6, 12, and 24 months postoperatively. Additionally, studies in the early mobilization group showed that tendon thickness gradually increased at 3, 6, and 12 months but tended to decrease at 24 months, possibly reflecting continuous functional remodeling of the induced tissue [15]. The delayed mobilization group showed a trend of decreasing tendon thickness over time, suggesting maturation and functional remodeling of new tissue, ultimately achieving or approaching the anatomical state of normal tendon [13]. For ASES scores, all groups showed significant improvement postoperatively compared to preoperative values. At 3 months, the early mobilization group had higher scores than the delayed mobilization group (52.0 vs. 29.6), while at 12 months the delayed mobilization group had higher functional scores than the early mobilization group (84.0 vs. 78.0). For Constant scores, at 3 months the delayed mobilization group had higher scores than the early mobilization group (85.67 vs. 44.0). Comprehensive analysis suggested no significant difference between early and delayed mobilization in terms of postoperative functional recovery or structural healing.

### 3.6 Safety Evaluation of the Resorbable Bio‐Inductive Collagen Implant

Seven incidences of implant‐related infection were reported in four of the included studies [17, 40, 42, 47]. Two incidences of implant‐related shoulder intermittent pain were reported in two of the included studies [39, 47]. One incidence of implant‐related increase in subacromial fluid was reported in one of the included studies [39]. The reported management measures included removal of implants, debridement, irrigation and drainage, antibiotic therapy, and ultrasound‐guided aspiration of effusion. After interventions, all cases reported recovery or remission [17, 39, 40, 47]. No implant‐related serious adverse events were reported (see Table 2 and Appendix S4).

### 3.7 Publication Bias and Sensitivity Analysis

This review included 17 studies, of which only 5 series were meta‐analyzed. Due to the limited number of included studies, publication bias or sensitivity analysis was not performed.

## 4 Discussion

### 4.1 Summary of Evidence

A total of 17 studies were included in the review, involving 1239 patients (sex was identified for 58.4% male and 41.6% female patients). The average follow‐up time was 12–60.3 months, and the follow‐up completion rate was above 80.0%.

We performed a quantitative meta‐analysis of the results of five case series. The findings showed that the resorbable bio‐inductive collagen implant had a good healing effect for rotator cuff injuries. Compared with the baseline measurements, the postoperative tendon thickness of the patients increased significantly at 3, 6, 12, and 24 months, and the postoperative shoulder function ASES and Constant scores at 6, 12, and 24 months were significantly better than the baseline. These findings were consistent with the studies of Thon, Stephen, and Schlegel et al. [7, 39].

In terms of safety, a total of seven incidences of implant‐related infection (out of 1256 implants) [17, 40, 42, 47], two incidences of implant‐related shoulder intermittent pain [39, 47], and one incidence of implant‐related increase in subacromial fluid [39] were reported in the included studies. No implant‐related serious adverse events were reported.

### 4.2 Safety of Resorbable Bio‐Inductive Collagen Implants in Repairing Rotator Cuff Injuries

Implanted medical devices have become an integral part of modern medicine, with widespread use accompanied by various adverse events [50]. After discussing with clinicians and experts of biomaterials and evidence‐based medicine, all adverse events reported in the literature were collected, but only implant‐related (serious) adverse events were discussed. See Section 2 for the definition of medical device‐related (serious) adverse events.

Infection is the most common adverse event in the application of medical devices [50]. In these included studies, seven infections were reported: specifically, two deep infections (reported by the authors) [40], one local superficial infection [47], and four infections with unclear status due to incomplete reporting [17, 40, 42]. The reported intervention measures included removal of implants, debridement, irrigation and drainage, and antibiotic treatment. All reported recovery or remission after interventions [9, 40, 47]. One of the case series reported positive deep shoulder infections for bacterial cultures obtained from deep glenohumeral joint or deltoid muscle specimens [51]. The incidence of deep infection after shoulder surgery is generally low. A cohort study [52] showed that the incidence of reoperation due to deep infection was only 0.1%. For rotator cuff repair procedures, however, the incidence was slightly higher (0.2%). The findings of this study are consistent with previous studies. Kwon et al. [53] suggested that incisional surgical site infections should be treated as early as possible with aggressive debridement and targeted antibiotic treatment. Suture anchors should be retained, the deltoid muscle reconnected to the acromion as much as possible, and the rotator cuff re‐repaired. McIntyre et al. [40] basically suggested the same interventions, reporting that if the infection was under control, then the problem was solved. Although the incidence of deep infection is low, it is still a potentially life‐threatening complication. For instance, Mirzayan et al. [54] conducted a retrospective analysis on 13 patients with deep postoperative infection. Even with multiple interventional approaches including debridement, irrigation, drainage, suture removal, bursa/synovectomy/sinus resection, targeted antibiotic therapy, and use of muscle flaps to cover infected shoulder wounds, most patient outcomes remained poor, with permanent functional deficits in some patients. Therefore, although the incidence of deep infection after rotator cuff repair is low, this outcome and prognosis are still worthy of clinical attention. Factors related to the increased infection rate after rotator cuff repair must be explored and effective preventive strategies sought in future research [52].

Apart from infection, two cases of implant‐related pain [39, 47] and one case of implant‐related subacromial effusion [39] were reported in the included studies. All reported recovery or remission after appropriate management. Pain was managed with intravenous antibiotics injection, bridge repair, and removal of residual sutures and anchors [47]; and cortisone injection [39]. Subacromial effusion was treated with ultrasound‐guided aspiration [39]. According to the clinical practice guidelines for rotator cuff injuries [28], however, xenografts would increase the incidence of postoperative adverse reactions. The recommendation was mainly based on one high‐quality study [55], one moderate‐quality study [56], and two low‐quality studies [57, 58]. In terms of source materials, porcine small intestinal submucosa [55, 56, 58] and porcine xenogenic dermis [57] were used. Studies have shown that implants derived from porcine small intestinal submucosa and porcine xenogenic dermis have strong immunogenicity, which is prone to adverse clinical outcomes, such as inflammation [59]. Resorbable bio‐inductive collagen implants, however, are composed of type I collagen derived from bovine Achilles tendon. Studies have shown that resorbable bio‐inductive collagen implants have good biocompatibility [7, 9] and no adverse inflammatory reactions [2, 3]. In terms of the types of adverse events that occurred, although the four studies included in the guidelines reported graft‐related adverse events, the implants used were not the resorbable bio‐inductive collagen implant, which is the focus of this study. Therefore, the safety of the resorbable bio‐inductive collagen implant is generally satisfactory. Meanwhile, the interim results of the latest randomized controlled trial also showed that there was no difference in complications between the experimental group (resorbable bio‐inductive collagen implant) and the control group (arthroscopic transosseous equivalent rotator cuff repair) (*p* > 0.05) [14]. If more detailed safety information is provided by the authors in this research project, it will help with a fuller understanding of the efficacy and safety of resorbable bio‐inductive collagen implants.

### 4.3 Efficacy of Resorbable Bio‐Inductive Collagen Implants in Repairing Rotator Cuff Injuries

#### 4.3.1 Tendon Thickness

Tendon thickness is an important outcome measure reflecting rotator cuff repair. The results of the meta‐analysis showed that the tendon thickness of patients with rotator cuff tear at 3, 6, 12, and 24 months after the resorbable bio‐inductive collagen implant was significantly increased compared with the baseline (*p* < 0.05). This is consistent with previous results [13]. This may be related to the fact that type I collagen could promote the early remodeling of the injured tendon and the implant has a high‐porosity structure (85% porosity), low elastic modulus, and a collagen fiber design consistent with the original tendon arrangement [60, 61, 62]. However, it should be noted that for patients with partial thickness tears and younger than 60 years old, although resorbable bio‐inductive collagen implants showed good curative effect (*p* < 0.05) with significantly increased postoperative tendon thickness, the results were from only two studies, both with small sample sizes (total sample size = 46) [15, 39]. For patients with full‐thickness tears and aged ≥ 60 years, one study (*n* = 104) reported a significant increase in tendon thickness at 24 months after surgery compared with the baseline (*p* < 0.05). However, due to the lack of early postoperative evidence, the exact effect of the resorbable bio‐inductive collagen implant was undetermined in patients with full‐thickness tears and aged ≥ 60 years.

High‐quality evidence supports the use of MRI, MRA, and ultrasound as effective clinical adjuncts in identifying rotator cuff tears, with MRI considered the best method for obtaining clear images of soft tissues [63, 64]. Of the three included studies that assessed tendon thickness, they were all measured with MRI. In two of these studies, the interpretation of the MRI results was assessed by a single radiologist and blinded. This method helped obtain objective and reliable data and reduce the measurement bias caused by the subjectivity of the MRI radiologist [15, 39]. The other study, however, did not report whether the clinician who interpreted the MRI results was blinded. This is not conducive to the accurate evaluation of the performance bias of the study based on the reported information [47]. The implementation of blinding is an important means to reduce measurement and implementation bias in clinical trials. It is therefore recommended that in future research, blinding should be implemented as much as possible during the trials to reduce bias, and detailed information should be reported to improve the transparency of the research [65].

#### 4.3.2 Physical Function

Shoulder joint dysfunction is one of the main clinical manifestations of rotator cuff injuries, and the improvement of dysfunction is the focus of treatment for patients with rotator cuff tendinopathy [66]. The functional limitation in patients with rotator cuff tears is mainly due to tendon injury. Histological studies show collagen structural degeneration, fat and blood vessels, and inflammatory cytokine infiltration in rotator cuff tendinopathy [67]. The results of the meta‐analysis showed that the shoulder joint function scores of patients at 6, 12, and 24 months after resorbable bio‐inductive collagen implants were significantly increased compared with the baseline. The dysfunction and shoulder pain were also significantly improved, which may be related to the promotion of tendon regeneration and neovascularization [7].

The American Shoulder and Elbow Surgeons Standardized Shoulder Assessment Form (ASES) score is an effective 100‐point scale that consists of two dimensions: 50% for pain and 50% for activities of daily living. The Constant score is a four‐item scale to evaluate shoulder joint function, recommended by the European Society of Shoulder and Elbow Surgery, weighted 15% for pain, 20% for activities of daily living, 40% for shoulder joint mobility, and 25% for muscle strength [68, 69]. Despite the wide use, however, the two scales are not the gold standard for evaluating shoulder joint function [29]. Given that the final ASES score is the sum score of pain and function, it is not conducive to evaluating the exact degree of functional limitation [70, 71]. For the Constant score, the 40% weight of shoulder joint mobility is quite heavy, despite no strong correlation between shoulder joint mobility and shoulder function shown in previous studies [72, 73]. Therefore, some scholars believe that these two scales are general evaluation tools for shoulder function rather than those for disease‐specific quality of life [74]. The patient perception of change in health status is the most important indicator of treatment success, and the inclusion of more patient‐centered outcome measures is critical to assess postoperative shoulder function in patients [28, 72]. Take the Western Ontario Rotator Cuff Index (WORC) quality of life assessment tool as an example. This tool is a strictly designed quality of life evaluation scale specific for patients with rotator cuff diseases. It contains 21 questions under five domains (physical symptoms, work, sports or recreational activities, lifestyle, and emotions). The WORC can be adopted as the main measurement tool to evaluate the prognosis of patients with rotator cuff injuries [28].

The difference in the implementation and interpretation of the scales and the lack of blinding of evaluators may be potential sources of bias. Studies have shown that compared with the original Constant evaluation scheme, there are different interpretations of the Constant scores by European users (or units). This is especially true in standardized evaluations, such as the way of asking patients and the guidance for test actions [75]. Among the included studies, none reported the detailed methods and standards for the scale administration, nor did they report whether the evaluators conducted a consistency test before assessing patients or whether they were blinded during the test. This is not conducive to the evaluation of the bias, especially for multi‐center clinical studies. Bankes et al. [76] proposed a comprehensive description of shoulder range of motion and muscle strength measurements with the Constant score. Unfortunately, their research results have not been successfully translated into clinical practice [75]. When future researchers select evaluation tools with items of controversial or uncertain methods, they should describe the corresponding evaluation methods in the manuscript and make decisions based on convincing prior evidence. Blinding and complete reporting of relevant information will help readers understand the research process, assess the risk of bias, and rate the quality of the results.

High‐quality evidence suggests that, for patients with small to moderate full‐thickness rotator cuff tears who undergo arthroscopic rotator cuff repair, the postoperative clinical outcomes of early or delayed mobilization are associated with patient‐reported outcome (PRO) scores [28]. Among the included studies, only one addressed the postoperative mobilization time [15]: Less than 1 week after the operation (i.e., early mobilization), the suspension immobilization was stopped and the patient gradually transitioned from passive to assisted active and active range of motion within the tolerance threshold. The study showed that the resorbable bio‐inductive collagen implant had good tissue induction and could significantly improve patient function. However, due to a lack of controls and incomplete reporting, it is difficult to rate the efficacy of resorbable bio‐inductive collagen implants on postoperative mobilization time, and if so, to what extent, and/or when the best postoperative mobilization time window is. Although the clinically significant postoperative mobilization time is easy to quantify, it is recommended to use more realistic and objective outcome measures when evaluating exercise load (such as absolute load and cyclic load). It will help clarify the effects of different rehabilitation programs on different types of rotator cuff tears and fixation methods in a shorter study period (e.g., 12 weeks) [28].

### 4.4 Potential Bias in the Review Process

Quantitative analysis was conducted on case series studies without a control group, which limits the quality of evidence. The included studies reported only limited basic patient information, without adequate demographic data such as age, sex, tear degree and type, and postoperative rehabilitation programs for subgroup analysis. This has limited the in‐depth analysis of the scope of application of resorbable bio‐inductive collagen implants. Two studies [17, 18] did not provide the necessary data for analysis, and one study [41] reported unclear information, making it impossible to judge whether the data were sufficient for data analysis. For the above studies, we tried to contact the authors to obtain raw data. Unfortunately, we did not obtain the necessary data for performing quantitative meta‐analysis of these studies.

## 5 Conclusion

Current evidence shows that resorbable bio‐inductive collagen implants are effective and safe in repairing rotator cuff injuries. The age of the patient may be an important factor affecting its efficacy. The impact of tear size and postoperative mobilization time on its efficacy needs to be clarified through in‐depth clinical research in the future. As far as the clinical application of the implant is concerned, future researchers should focus on the efficacy and safety of the implant on specific subgroups (e.g., degree and type of rotator cuff tear, applicable population, and age). It might be worthwhile to explore the postoperative rehabilitation program, with patient‐centered outcome measures. Additionally, future researchers should strictly control the risk of bias in research design and implementation, and improve the transparency of reporting. Blinding should be implemented as much as possible, and implementation details should be reported for the evaluation of subjective outcome measures to ensure the internal and external validity of the results.

## Author Contributions

**Jiaxin Tian, Fengxing Ding:** conceptualization, methodology, data curation and writing – original draft; **Zhe Wang, Niu Muting, Chen Liu:** data curation and investigation; **Zipeng Ye, Huiang Chen, Caizhi Wu, Shaowei Yi:** formal analysis and validation; **Yubo Fan, Jinzhong Zhao, Shiyi Cao, Bin Ma:** writing – review and editing, supervision and project administration.

## Conflicts of Interest

The authors declare no conflicts of interest.

## Supporting information


**Appendix S1:** os70141‐sup‐0001‐Appendix1.docx.

**Appendix S2:** os70141‐sup‐0002‐Appendix2.xlsx.

**Appendix S3:** os70141‐sup‐0003‐Appendix3.xlsx.

**Appendix S4:** os70141‐sup‐0004‐Appendix4.docx.
