# Supplementary material for: Resorbable Bio‐Inductive Collagen Implant for Rotator Cuff Repair: What We Know, What We Need to Know, and the Path Forward
Source: Orthop Surg. 2025 Aug 8;17(9):2541–57. doi: 10.1111/os.70141 (PMC12404871; doi:10.1111/os.70141)
Supplement: Supplementary file 1 — Appendix S1: os70141‐sup‐0001‐Appendix1.docx. [file OS-17-2541-s002.docx]

# Searching strategies and records

**Initial search：2022.09**

**PubMed：**

(Regeneten bio-inductive collagen scaffold[Text Word]) OR (Regeneten implant[Text Word]) OR (biological augmentation[Title/Abstract]) OR (collagen implant[Title/Abstract]) OR (bioabsorbable collagen implant[Title/Abstract]) OR (bioinductive collagen patch[Title/Abstract]) OR (bioabsorbable collagen implant[Title/Abstract]) OR (REGENETEN Collagen Implant[Text Word]) OR (Porous Collagen Implant[Title/Abstract]) OR (bio-inductive scaffold implant[Title/Abstract]) OR (REGENETEN Bioinductive Implant[Text Word]) OR (collagen scaffold[Title/Abstract]) OR (REGENETEN[Text Word]) [**1,625**](https://pubmed.ncbi.nlm.nih.gov/?term=(Regeneten+bio-inductive+collagen+scaffold%5bText+Word%5d)+OR+(Regeneten+implant%5bText+Word%5d)+OR+(biological+augmentation%5bTitle/Abstract%5d)+OR+(collagen+implant%5bTitle/Abstract%5d)+OR+(bioabsorbable+collagen+implant%5bTitle/Abstract%5d)+OR+(bioinductive+collagen+patch%5bTitle/Abstract%5d)+OR+(bioabsorbable+collagen+implant%5bTitle/Abstract%5d)+OR+(REGENETEN+Collagen+Implant%5bText+Word%5d)+OR+(Porous+Collagen+Implant%5bTitle/Abstract%5d)+OR+(bio-inductive+scaffold+implant%5bTitle/Abstract%5d)+OR+(REGENETEN+Bioinductive+Implant%5bText+Word%5d)+OR+(collagen+scaffold%5bTitle/Abstract%5d)+OR+(REGENETEN%5bText+Word%5d)&sort=relevance&size=100&ac=no)

**Web of science：**

TS=(("Regeneten bio-inductive collagen scaffold") OR ("Regeneten implant") OR ("biological augmentation") OR ("collagen implant") OR ("bioabsorbable collagen implant") OR ("bioinductive collagen patch") OR ("bioabsorbable collagen implant") OR ("REGENETEN Collagen Implant") OR ("Porous Collagen Implant") OR ("bio-inductive scaffold implant") OR ("REGENETEN Bioinductive Implant") OR ("collagen scaffold") OR (REGENETEN)) **2,986**

**Embase:**

("Regeneten bio-inductive collagen scaffold") OR ("Regeneten implant") OR ("biological augmentation") OR ("collagen implant") OR ("bioabsorbable collagen implant") OR ("bioinductive collagen patch") OR ("bioabsorbable collagen implant") OR ("REGENETEN Collagen Implant") OR ("Porous Collagen Implant") OR ("bio-inductive scaffold implant") OR ("REGENETEN Bioinductive Implant") OR ("collagen scaffold") OR (REGENETEN) **3,082**

**The Cochrane library：**

("Regeneten bio-inductive collagen scaffold") OR ("Regeneten implant") OR ("biological augmentation") OR ("collagen implant") OR ("bioabsorbable collagen implant") OR ("bioinductive collagen patch") OR ("bioabsorbable collagen implant") OR ("REGENETEN Collagen Implant") OR ("Porous Collagen Implant") OR ("bio-inductive scaffold implant") OR ("REGENETEN Bioinductive Implant") OR ("collagen scaffold") OR (REGENETEN)  **186**

**Update retrieval 2023.06**

**PubMed：**

(Regeneten bio-inductive collagen scaffold[Text Word]) OR (Regeneten implant[Text Word]) OR (biological augmentation[Title/Abstract]) OR (collagen implant[Title/Abstract]) OR (bioabsorbable collagen implant[Title/Abstract]) OR (bioinductive collagen patch[Title/Abstract]) OR (bioabsorbable collagen implant[Title/Abstract]) OR (REGENETEN Collagen Implant[Text Word]) OR (Porous Collagen Implant[Title/Abstract]) OR (bio-inductive scaffold implant[Title/Abstract]) OR (REGENETEN Bioinductive Implant[Text Word]) OR (collagen scaffold[Title/Abstract]) OR (REGENETEN[Text Word]) Filters: from 2022 - 2023 **156**

**Web of science：**

TS=(("Regeneten bio-inductive collagen scaffold") OR ("Regeneten implant") OR ("biological augmentation") OR ("collagen implant") OR ("bioabsorbable collagen implant") OR ("bioinductive collagen patch") OR ("bioabsorbable collagen implant") OR ("REGENETEN Collagen Implant") OR ("Porous Collagen Implant") OR ("bio-inductive scaffold implant") OR ("REGENETEN Bioinductive Implant") OR ("collagen scaffold") OR (REGENETEN)) 2022-09-01 to 2023-06-21 (publication date) **104**

**Embase:**

('regeneten bio-inductive collagen scaffold' OR 'regeneten implant' OR 'biological augmentation' OR 'collagen implant'/exp OR 'collagen implant' OR 'bioinductive collagen patch' OR 'bioabsorbable collagen implant' OR 'regeneten collagen implant' OR 'porous collagen implant' OR 'bio-inductive scaffold implant' OR 'regeneten bioinductive implant' OR 'collagen scaffold'/exp OR 'collagen scaffold' OR 'regeneten'/exp OR regeneten) AND [2022-2023]/py **243**

**The Cochrane library：**

("Regeneten bio-inductive collagen scaffold") OR ("Regeneten implant") OR ("biological augmentation") OR ("collagen implant") OR ("bioabsorbable collagen implant") OR ("bioinductive collagen patch") OR ("bioabsorbable collagen implant") OR ("REGENETEN Collagen Implant") OR ("Porous Collagen Implant") OR ("bio-inductive scaffold implant") OR ("REGENETEN Bioinductive Implant") OR ("collagen scaffold") OR (REGENETEN) **14**
