# Supplementary figures and images for: Resorbable Bio‐Inductive Collagen Implant for Rotator Cuff Repair: What We Know, What We Need to Know, and the Path Forward
Source: Orthop Surg. 2025 Aug 8;17(9):2541–57. doi: 10.1111/os.70141 (PMC12404871; doi:10.1111/os.70141)

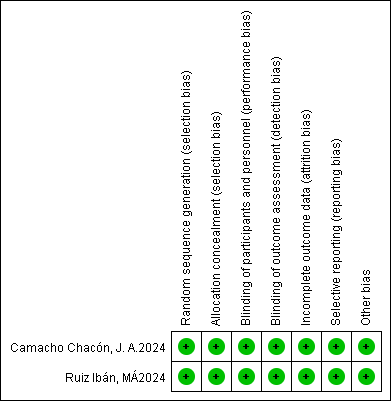
 RCT


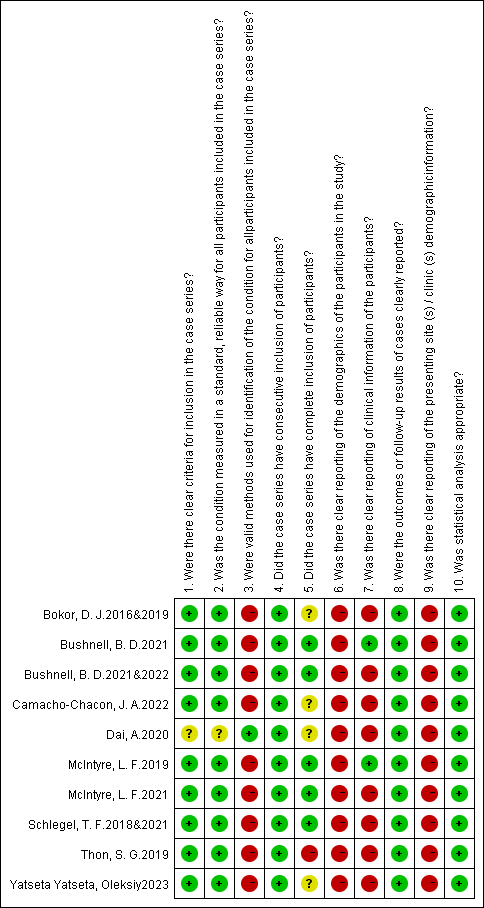
 case series


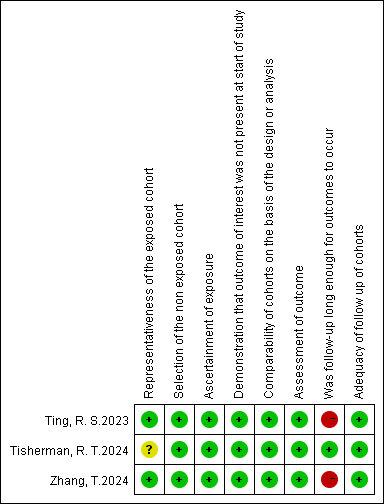
cohort study

Supplement: Supplementary file 4 — Appendix S4: os70141‐sup‐0004‐Appendix4.docx. [file OS-17-2541-s001.docx]
